# Supplementary material for: Relationship between Visual Perception and Microstructural Change of the Superior Longitudinal Fasciculus in Patients with Brain Injury in the Right Hemisphere: A Preliminary Diffusion Tensor Tractography Study
Source: Diagnostics (Basel). 2020 Aug 27;10(9):641. doi: 10.3390/diagnostics10090641 (PMC7555244; doi:10.3390/diagnostics10090641)
Supplement: Supplementary file 1 [file diagnostics-10-00641-s001.pdf]

Supplimentary Table S1. MVPT Sheet

Contents	Item				
Visual discrimination	1	**A**	B	C	D
	2	A	B	**C**	D
	3	A	B	**C**	D
	4	A	B	C	**D**
	5	A	**B**	C	D
	6	A	**B**	C	D
	7	A	B	C	**D**
	8	A	**B**	C	D
Figure-gorund discrimination	9	A	**B**	C	D
	10	A	B	C	**D**
	11	**A**	B	C	D
	12	**A**	B	C	D
	13	A	**B**	C	D
Visual memory	14	A	**B**	C	D
	15	A	B	C	**D**
	16	**A**	B	C	D
	17	**A**	B	C	D
	18	**A**	B	C	D
	19	A	B	**C**	D
	20	A	B	C	**D**
	21	**A**	B	C	D
Visual closure	22	A	**B**	C	D
	23	**A**	B	C	D
	24	A	**B**	C	D
	25	A	B	C	**D**
	26	A	**B**	C	D
	27	A	B	C	**D**
	28	**A**	B	C	D
	29	A	B	C	**D**
	30	A	B	**C**	D
	31	A	B	C	**D**
	32	**A**	B	C	D
Spatial relation ship	33	A	**B**	C	D
	34	A	B	**C**	D
	35	A	B	**C**	D
	36	A	**B**	C	D
Raw score					
Left/Right response					

Processing time	

Note: Bold, right answer
